# Clinical Results of Minimally Invasive Spine Stabilization for the Management of Metastatic Spinal Tumors Based on the Epidural Spinal Cord Compression Scale

**DOI:** 10.1155/2018/1258706

**Published:** 2018-11-08

**Authors:** Hiroshi Uei, Yasuaki Tokuhashi, Masafumi Maseda, Masahiro Nakahashi, Hirokatsu Sawada, Koji Matsumoto, Hiroyuki Miyakata, Hirotoki Soma

**Affiliations:** Department of Orthopaedic Surgery, Nihon University School of Medicine, 30-1 Oyaguchi Kami-cho, Itabashi-ku, Tokyo 173-8610, Japan

## Abstract

**Purpose:**

Minimally invasive spine stabilization (MISt) using percutaneous pedicle screws plays a significant role in palliative surgery for metastatic spinal tumors. However, few studies have investigated surgical outcomes based on the epidural spinal cord compression scale (ESCCS). The purpose of this study was to examine outcomes of metastatic spinal tumors as evaluated by ESCCS in patients treated by MISt.

**Methods:**

The subjects were 56 patients who underwent MISt for metastatic spinal tumors, including 34 patients with ESCCS 2 or milder (group A) and 22 patients with ESCCS 3 (group B). We analyzed baseline characteristics, perioperative factors and clinical results such as postoperative survival time, neurological outcomes, Barthel Index for activities of daily living (ADL), visual analogue scale (VAS), and the rate of discharge to home.

**Results:**

The baseline age (P=0.07), tumor diagnosis (P=0.23), spinal level of compression (P=0.35), American Spinal Injury Association classification (P=0.49), revised Tokuhashi score (P=0.92), spinal instability neoplastic score (P=0.28), VAS (P=0.35), Barthel Index (P=0.07), American Society of Anesthesiologists physical status classification (P=0.76), and type of surgery (P=0.40) did not differ significantly between the two groups. The median postoperative survival time did not differ significantly between the groups (12.0 versus 15.0 months, P=0.60). Neurological improvement by at least 1 grade or maintenance of grade E was favorable in group A. Patients in group A had less posterior decompression (P=0.006), a higher rate of chemotherapy (P=0.009), a higher postoperative Barthel Index (P=0.04), and a higher rate of discharge to home (P=0.01) and no patients died in the hospital (P=0.004).

**Conclusions:**

No significant difference was noted in the postoperative survival time between the 2 groups. Patients in the ESCCS 2 or milder group had favorable neurological improvement, higher rates of chemotherapy, better postoperative ADL, and the higher rate of discharge to home.

## 1. Introduction

The majority of metastatic spinal tumors are cancer metastasis, a systemic disease for which treatment is limited [1–4]. However, with advances in cancer therapy, the survival time for metastatic cancer patients has been extended for some cancer types [5, 6] and has subsequently increased the amount of symptomatic spinal metastasis cases [7]. Metastatic spinal tumors destroy the spine, collapse spinal support, and invade and compress the spinal cord and cauda equina, inducing pain, paralysis, and disturbance of activities of daily living (ADL). Evaluation methods of the grade of nerve compression associated with metastatic spinal tumors include the epidural spinal cord compression scale (ESCCS) based on T2-weighted axial magnetic resonance imaging (MRI) [8]. This scale employs a 6-grade evaluation method where 0 represents a tumor staying in the vertebra, 1a represents an epidural impingement without deformation of the thecal sac, 1b represents a tumor compressing the thecal sac, and 1c represents a deformation of the thecal sac with spinal cord abutment in the absence of spinal cord compression. ESCCS 2 represents a tumor compressing the spinal cord, but cerebrospinal fluid is visualized around the cord, and ESCCS 3 represents a tumor compressing the spinal cord without visualization of cerebrospinal fluid around the cord. We previously investigated the severity and progression of paralysis based on the spinal cord level with paralysis using ESCCS in patients with metastatic spinal tumors. The severity of paralysis differed depending on the spinal cord level, but no correlation was noted between the severity of paralysis and ESCCS at any level [9]. Quraishi et al. divided patients into 2 groups, ESCCS 1c or milder without spinal cord compression or ESCCS 2 and 3 with spinal cord compression, and compared the outcome of surgical decompression (+/- stabilization) [10]. No significant difference was noted in the improvement of postoperative survival or neurological status, but many cases remained Frankel grade E in the ESCCS 1c or milder group after surgery.

**Figure 1:**
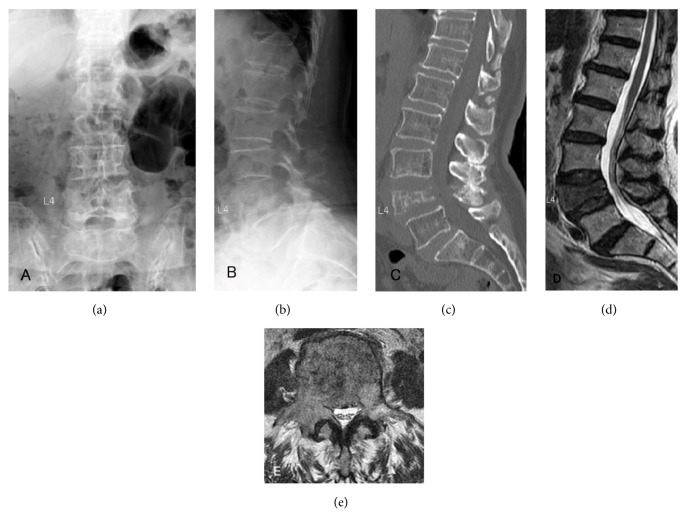
An 80-year-old woman with metastasis of unknown origin to the L4 lumbar vertebrae. The preoperative ASIA classification was E. The modified Tokuhashi score was 11. (a) Posteroanterior view on preoperative radiography. (b) Lateral view on preoperative radiography. (c) Sagittal view on preoperative plain CT. (d) Sagittal view on preoperative T2-weighted MRI. (e) Axial view at the L4 lumbar vertebrae on preoperative T2-weighted MRI.

**Figure 2:**
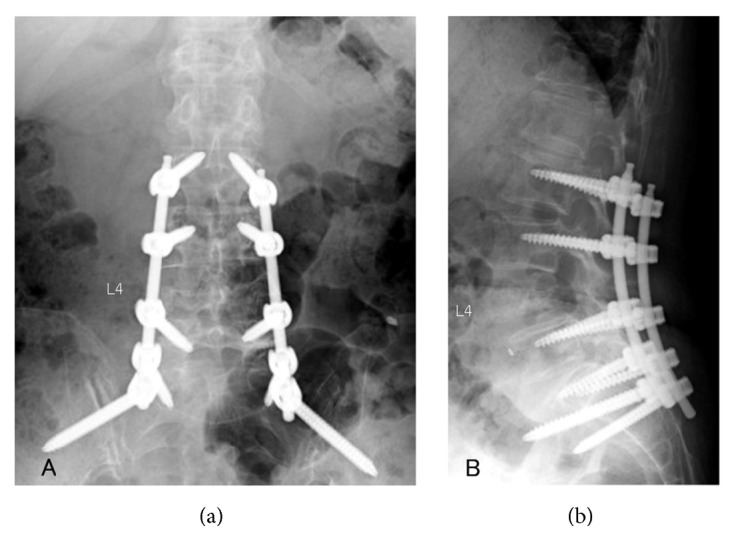
Immediately after minimally invasive spine stabilization without decompression (L2-S2AI) and biopsy of the L4 lumbar vertebrae through the right pedicle were performed. (a) Posteroanterior view on radiography. (b) Lateral view on radiography.

**Figure 3:**
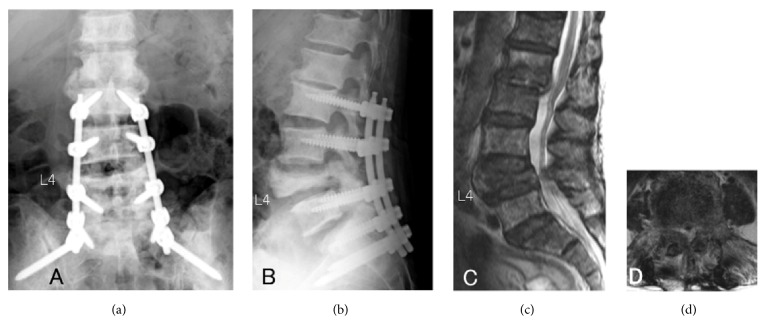
26 months after minimally invasive spine stabilization. (a) Posteroanterior view on radiography. (b) Lateral view on radiography. (c) Sagittal view on T2-weighted MRI. (d) Axial view of the L4 lumbar vertebrae on T2-weighted MRI.

Surgery for metastatic spinal tumors is palliative in many cases, but surgical stress cannot be neglected for patients with a short predicted survival [11]. For surgical treatment for metastatic spinal tumor patients, minimization of surgical stress has been attempted, such as minimally invasive spine stabilization (MISt) using percutaneous pedicle screws (PPS) (Figures 1–3) [12–21] and balloon kyphoplasty [22]. Minimization of palliative surgery stress and multidisciplinary treatment has improved ADL, facilitated discharge to home, and enabled postoperative adjuvant therapy at a high rate [23]. MISt is more useful to minimize the stress of surgery for metastatic spinal tumors than for spinal degenerative disease, but no study has investigated the surgical outcomes of MISt in cases of different grades of spinal cord compression, although evaluation of the outcomes of conventional palliative surgical decompression (+/- stabilization) in cases of different grades of spinal cord compression has been reported [10]. In this study, patients treated by MISt for metastatic spinal tumors were divided into 2 groups based on the grade of spinal cord compression and differences in the treatment outcomes were retrospectively investigated.

## 2. Patients and Methods

### 2.1. Patient Population

We performed a retrospective review using prospectively collected data from 100 consecutive patients who underwent palliative surgery for metastatic spinal tumors between January 2012 and December 2017 at our institution. MISt with PPS for metastatic spinal tumors [15, 16, 23] was indicated for intractable pain due to spinal instability or threat of instability defined by the spinal instability neoplastic score (SINS) [24], spinal paralysis, such as any change in the motor examination, or radiation-resistant cancer such as kidney cancer or thyroid cancer. We excluded patients for whom total en bloc spondylectomy was indicated for spinal levels at the occipital to the cervical region; it was impossible to confirm the pedicle of the vertebral arch under fluoroscopy or impossible to insert PPS who were treatable with balloon kyphoplasty, who were treatable with posterior decompression alone, or who had a life expectancy less than 6 months and were responsive to narcotic analgesics or markedly responsive to radiotherapy, poor general condition (Karnofsky performance status 3 or poorer), or a reduced will to live. When PPS was unable to be inserted, we performed conventional posterior decompression and fixation surgery. In total, 56 patients met the inclusion for this study. After approval by the Nihon University Hospital Joint Institutional Review Board, all participants provided written informed consent. From our cohorts, we divided the patients into 2 groups: low grades of epidural spinal cord compression scale (ESCCS) 0, 1a, 1b, 1c, and 2 (group A: n=34) or high grade of ESCCS 3 (group B: n=22) [8].

### 2.2. Outcome Evaluation

Baseline characteristics included age, sex, metastatic tumor diagnosis, main spinal level of compression, preoperative American Spinal Injury Association (ASIA) classification [25], revised Tokuhashi score [3], SINS [24], ESCCS, preoperative visual analogue scale (VAS) for pain, preoperative Barthel Index for ADL [26], American Society of Anesthesiologists (ASA) physical status classification, and type of surgery (emergency: within 24 hours of presentation, urgent: within 1-3 days of presentation, and scheduled: greater than 3 days from presentation). Perioperative factors included operation time, intraoperative blood loss, blood transfusion rate, number of levels fused, incidence of perioperative complications, and use of additional adjuvant therapy (chemotherapy, radiotherapy, or introduction of bone modifying agent (BMA) therapy). Clinical data included postoperative survival time, grade of postoperative paralysis, paralysis improvement by one or more grades, or maintenance of grade E on the ASIA classification, Barthel Index (postoperative maximum score), VAS at 2 weeks after surgery, postoperative course (discharge to home, transfer to hospice, and in-hospital death), and revision surgery due to tumor enlargement at the surgically treated level. Regarding postoperative adjuvant therapy, radiotherapy was performed after surgery except in the case of radiation-resistant cancer, provided that it was not inconvenient for treatment of the primary cancer. Discharge to home was selected if the Barthel Index of the patient was over 70 or availability of sufficient care by family members was expected for those with a Barthel Index lower than 70. Statistical analysis was performed using IBM SPSS Statistics version 25 (IBM Corporation, Armonk, NY, USA). The Kaplan-Meier method was used to estimate postoperative survival, and survival curves were compared using the log-rank test. The paired t-test and Mann-Whitney* U* test were used for continuous variables, and the *χ*^2^ test was applied for categorical data. In all cases, the significance level was set at* P* < 0.05.

## 3. Results

A total of 56 patients, including 34 patients in group A and 22 patients in group B, were enrolled in the study (Table 1). The mean ages at the time of surgery were 69.9 and 63.4 years in groups A and B, respectively (P=0.07). Group A included 11 males (32.4%) and 23 females (67.6%), and group B included 2 males (9.1%) and 20 females (90.9%) (P=0.04). The primary tumors were in the lung in 10 (29.4%) and 2 (13.6%), prostate in 2 (5.9%) and 5 (22.7%), liver in 5 (14.7%) and 5 (22.7%), gastric in 1 (2.9%) and 2 (9.1%), thyroid in 2 (5.9%) and 1 (4.5%), and others in 9 (26.4%) and 5 (22.7%), in groups A and B, respectively (P=0.64). The primary tumors were in the kidney in 4 (11.8%) only in group A and breast in 2 (9.1%) only in group B. The main spinal levels of compression were the thoracic and lumbar spine in 15 (44.1%) and 19 (55.9%) patients in group A, respectively, and 7 (31.8%) and 15 (68.2%) patients in group B (P=0.35), respectively. The ASIA classification (P=0.49), revised Tokuhashi score (P=0.92), SINS (P=0.28), VAS scores (P=0.35), preoperative Barthel Index (P=0.07), ASA physical status classification (P=0.76), and type of surgery (P=0.40) did not differ between the two groups. Regarding the mean revised Tokuhashi score, there was also no significant difference between group A (6.2 ± 2.8) and group B (6.8 ± 2.8) (P=0.50). In group A, the ESCCS was 0 in 3 (8.8%), 1a in 4 (11.8%), 1b in 1 (2.9%), 1c in 3 (8.8%), and 2 in 23 (67.6%). In group B, the ESCCS was 3 in 22 (100%) (P<0.001).

There were no significant differences in operative time (P=0.24), blood loss (P=0.19), blood transfusion rates (P=0.29), number of levels fused (P=0.53), or incidence of perioperative complications (P=0.23) between the two groups (Table 2). Patients in group B underwent posterior decompression more frequently (P=0.006). Rates of radiotherapy (P=0.56) and BMA therapy (P=0.36) did not differ significantly between groups A and B. Significantly more patients in group A underwent adjuvant chemotherapy (P=0.009).

**Figure 4:**
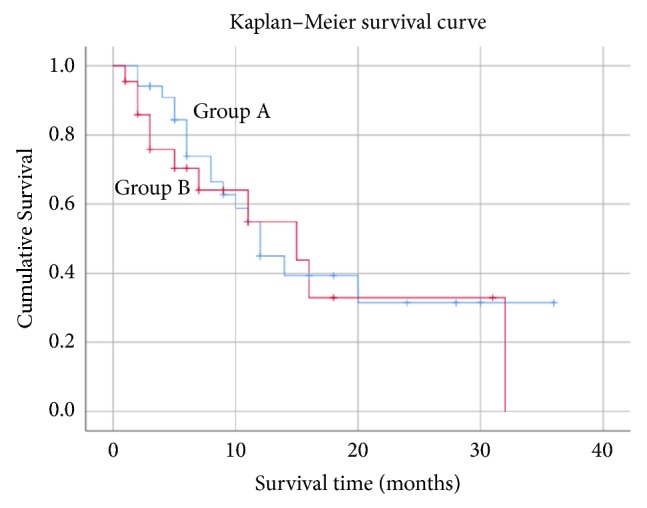
Kaplan-Meier survival curves for patients with metastatic spinal tumors treated by minimally invasive spine stabilization in group A (ESCCS 0, 1, 2) and group B (ESCCS 3). The median survival time was 12.0 months (95% confidence interval: 9.1-14.8) in group A and 15.0 months (95% confidence interval: 3.9–26.0) in group B, with no significant difference between the two groups (P=0.60).

The median postoperative survival time determined using the Kaplan-Meier method was 12.0 months (95% confidence interval (CI): 9.1-14.8) in group A and 15.0 months (95% CI: 3.9–26.0) in group B, with no significant difference between the two groups (P=0.60) (Figure 4). The postoperative ASIA classification (P=0.15) and postoperative VAS scores (P=0.21) did not differ significantly between groups A and B. Neurological improvement by at least 1 grade or maintenance of grade E differed significantly between groups A (67.6%) and B (40.9%) (P=0.048) (Tables 3 and 4). The postoperative Barthel Index was 87.9 and 68.8 in groups A and B, respectively, with a significant difference between the two groups (P=0.04). Significantly more patients in group A were discharged to home (P=0.01), and no patients died in the hospital (P=0.004). The rate of revision surgery for local recurrence due to tumor enlargement at the treated level did not differ significantly between groups A and B (P=0.60).

## 4. Discussion

Bilsky et al. previously reported the superiority of the 6-grade ESCCS on T2-weighted axial imaging as a method to evaluate the grade of nerve compression by metastatic spinal tumors [8]. However, when we previously investigated the severity and progression of paralysis using the ESCCS, the severity of paralysis differed depending on the spinal cord level, and the severity of paralysis was not correlated with the ESCCS at any level [9]. Regarding the postoperative outcomes of tumors with different degrees of spinal cord compression, Quraishi et al. reported no significant difference in neurological improvement between ESCCS 1c or milder with no spinal cord compression group and ESCCS 2 or 3 with spinal cord compression group [10]. However, as the ESCCS 1c or milder group included many patients with Frankel grade E before surgery, the baseline characteristics were heterogeneous. Therefore, it is inappropriate to separate between ESCCS 1c or milder and 2 when grouping the patients. The patients in our study were divided at ESCCS 2 or milder and ESCCS 3 with high-grade spinal cord compression in order to prevent the baseline characteristics from being heterogeneous, especially preoperative neurological status, and there was no significant difference in the baseline characteristics excluding the sex and ESCCS. Oshima et al. reported that it was possible to predict the postoperative walking function from the circumferential ratio of cord compression (CRCC) on T2-weighted axial MRI in a retrospective study [27]. They found that functional ambulation was lost in accordance with CRCC in a quantitative manner, and when it exceeded more than 2/4, the decrease in functional prognosis was significant. In our study, neurological improvement by one or more grades or maintenance of grade E on the ASIA classification was noted in 23 (67.6%) and 9 (40.9%) patients in groups A and B, respectively (P=0.048), demonstrating that improvement was favorable in the ESCCS 2 or milder group. Regarding neurological recovery, there was a significant difference in the grade of spinal cord compression by metastatic spinal tumors between the 2 groups.

It remains still difficult to accurately predict the survival time for metastatic spinal tumor patients before surgery [2–5, 11], and the significance of palliative surgery for patients with a short predicted survival is controversial. In the present study, the percentages of patients with a revised Tokuhashi score of 8 or lower were 76.5% and 72.7% in groups A and B, respectively, and the means were 6.2 and 6.8, respectively, suggesting that the predicted survival time was shorter than 6 months for many patients. When palliative surgery is performed for patients with a predicted survival time shorter than 6 months, it is necessary to consider the benefits of surgery versus the risks of surgery-associated complications, and medical costs of surgery should also be discussed [11, 28, 29]. Invasive palliative surgery for metastatic spinal tumor patients may aggravate the general condition after surgery, and the opportunity to receive postoperative adjuvant therapy may be delayed or lost. However, in one previous report, surgery improved the ADL and increased the opportunity to receive postoperative adjuvant therapy, for which indirect prolonging of life can be expected [30]. Therefore, surgical stress by palliative surgery has recently been minimized [12–21]. Compared with conventional posterior decompression fusion surgery, the surgical wound from MISt using PPS is small, the operative time is short, intraoperative blood loss is low, and the wound heals rapidly, facilitating adjuvant therapy early after surgery [15, 16, 23]. In the present study, the median survival time after surgery determined using the Kaplan-Meier method was 12.0 months in group A (95% CI: 9.1-14.8) and 15.0 months (95% CI: 3.9-26.0) in group B (P=0.60). The median postoperative survival time was longer than the predicted survival time in both groups even though the grade of spinal cord compression was different, and this may have been related to the high rate of adjuvant therapy after surgery due to reduced surgical stress by MISt.

Of the patients who underwent MISt for metastatic spinal tumors, for those without posterior decompression, the operative time is shorter and blood loss is lower, further minimizing the invasiveness [16]. In the present study, posterior decompression was performed for 12 patients (35.3%) in group A, which was significantly fewer (P=0.006), but the operative time (P=0.24) and blood loss (P=0.19) were not significantly different, albeit slightly lower. On the other hand, postoperative chemotherapy was performed for 27 patients (79.4%) in group A, which was a significantly higher rate (P=0.009), and the mean postoperative Barthel Index was 87.9 in group A, being significantly higher (P=0.04), suggesting that postoperative recovery was favorable in group A. As no significant difference was noted in the postoperative survival time between the 2 groups, the difference in the surgical stress level was likely reflected in the postoperative ADL, leading to the significantly higher rate of discharge to home (87.9%) (P=0.01) and the absence of in-hospital death (P=0.004) in group A. Therefore, the influence of surgical stress due to posterior decompression may be negligible.

Most metastatic spinal tumors are cancer metastasis, a systemic disease for which treatment is limited [1–4]. Evaluation of the outcome of low-invasive palliative surgery for metastatic spinal tumors has focused only on the technical aspects, surgical stress, and short-term improvement of paralysis, and the postoperative survival time, ADL, and quality of life (QOL) were evaluated in only a few studies [15, 16, 18, 19, 23]. To maintain the QOL of cancer patients, it is important to continue adjuvant therapy, such as chemotherapy and radiotherapy, after surgery. The objectives of surgery for metastatic spinal tumor patients should include not only short-term improvement of paralysis but also improvement of ADL to enable discharge to home and continuation of postoperative adjuvant therapy at an outpatient clinic. Regarding palliative care for cancer patients close to death, the survival time of cancer patients is markedly longer for those who die at home rather than at the hospital [30]. In the present study, no significant difference was noted in the postoperative survival time between the 2 groups (P=0.60), but the rate of discharge to home was 87.9% in group A, being significantly higher (P =0.01). The mean preoperative Barthel index was higher in group A (67.7) than in group B (50.0), although there was no significant difference. Indeed, the preoperative ADL was better in group A than in group B, and the higher rate of discharge to home in group A might be influenced by the higher preoperative Barthel index. Therefore, MISt applied in the absence of spinal cord compression or in the presence of relatively mild spinal cord compression by the tumor improved postoperative ADL and led to higher rate of discharge to home.

There are several limitations in this study. First, this was a retrospective study. Symptoms of spinal cord compression vary with spinal levels and have a significant impact on the preoperative condition such as neurological status and Barthel index. We previously reported that the severity of paralysis was not correlated with ESCCS at any level [9]. In the current study, the preoperative main spinal levels of compression were the thoracic and lumbar spine in 15 (44.1%) and 19 (55.9%) patients in group A, respectively, and 7 (31.8%) and 15 (68.2%) patients in group B, respectively, and there was no significant difference between the groups. Furthermore, no significant difference was noted in the baseline characteristics excluding the sex and ESCCS, which may reduce the impact of the results. However, the preoperative rate of ASIA classification E was 20.6% in group A and 9.1% in group B, being higher in group A. Group A had the better preoperative Barthel Index. These variables might be confounders. Therefore, heterogeneity cannot be completely ruled out, as in the study reported by Quraishi et al. [10].

Second, we did not investigate the treatment outcome according to each ESCCS grade because of the small number of patients. Moreover, it is unclear whether it is appropriate to separate between ESCCS 2 and 3 when grouping patients. Bilsky et al. classified ESCCS 2 tumors compressing the spinal cord as a high grade from the viewpoint of radiotherapy [8]. Quraishi et al. employed the classification by Bilsky et al. [10]. In our previous study [9], ESCCS 2 or severer with paralysis at the C7-L1 level equaled C or severer on the ASIA classification [25], indicating that ESCCS 2 is a high grade. However, in the present study, posterior decompression was performed for 12 (35.3%) and 16 (72.7%) patients in groups A and B, respectively (P=0.006). Thus, for actual selection of the surgical procedure, there may be no problem with classifying groups A and B as low- and high-grade spinal cord compression, respectively.

Third, as the number of patients was small, investigation based on the involved organ was not possible, being a limitation of a single-center study. A large-scale investigation involving several institutions may be necessary.

## 5. Conclusions

Metastatic spinal tumor patients treated by MISt were divided into those with ESCCS 2 or milder spinal cord compression or those with ESCCS 3 severer spinal cord compression based on the preoperative grade of spinal cord compression, and the treatment outcomes were retrospectively investigated. No significant difference was noted in the baseline characteristics excluding the sex and ESCCS. Neurological improvement by at least 1 grade or maintenance of grade E was favorable in the ESCCS 2 or milder group. No significant difference was noted in the postoperative survival time between the 2 groups. In the ESCCS 2 or milder group, posterior decompression was performed for significantly fewer patients; postoperative chemotherapy was performed at a significantly higher rate. They had the better postoperative ADL and the higher rate of discharge to home.

## Figures and Tables

**Table 1:** Baseline characteristics of group A (ESCCS 0,1,2) and group B (ESCCS 3).

Characteristic		Group A (n=34)	Group B (n=22)	P value
Age at surgery, mean (SD), years		69.9 (8.1)	63.4 (15.1)	0.07

Sex, n (%)	Male	11 (32.4)	2 (9.1)	0.04
	Female	23 (67.6)	20 (90.9)	

Metastatic tumor diagnosis, n (%)	Lung	10 (29.4)	2 (13.6)	0.23
	Prostate	2 (5.9)	5 (22.7)	
	Liver	5 (14.7)	5 (22.7)	
	Gastric	1 (2.9)	2 (9.1)	
	Kidney	4 (11.8)	0	
	Breast	0	2 (9.1)	
	Thyroid	2 (5.9)	1 (4.5)	
	Others	9 (26.4)	5 (22.7)	
	Unknown	1 (8.3)	0	

Main spinal level of compression, n (%)	Thoracic	15 (44.1)	7 (31.8)	0.35
	Lumbar	19 (55.9)	15 (68.2)	

Preoperative ASIA classification, n (%)	A	0	1 (4.5)	0.49
	B	1 (2.9)	1 (4.5)	
	C	6 (17.6)	6 (27.3)	
	D	20 (58.8)	12 (54.5)	
	E	7 (20.6)	2 (9.1)	

Revised Tokuhashi score, n (%)	0-8	26 (76.5)	16 (72.7)	0.92
	9-11	7 (20.6)	5 (22.7)	
	12-15	1 (2.9)	1 (4.5)	

Spinal instability neoplastic score, n (%)	0-6	3 (8.8)	1 (4.5)	0.28
	7-12	30 (88.2)	18 (81.8)	
	13-18	1 (2.9)	3 (13.6)	

Epidural spinal cord compression scale, n (%)	0	3 (8.8)	0	<0.001
	1a	4 (11.8)	0	
	1b	1 (2.9)	0	
	1c	3 (8.8)	0	
	2	23 (67.6)	0	
	3	0	22 (100)	

Preoperative visual analogue scale, mean (SD)		5.5 (2.1)	6.1 (2.3)	0.35

Preoperative Barthel index, mean (SD)		67.7 (30.8)	50.0 (38.3)	0.07

ASA physical status classification, n (%)	1	2 (5.9)	2 (9.1)	0.76
	2	27 (79.4)	18 (81.8)	
	3	5 (14.7)	2 (9.1)	

Type of surgery, n (%)	Scheduled	32 (94.1)	19 (86.4)	0.40
	Urgent	2 (5.9)	1 (4.5)	
	Emergency	0	2 (9.1)	

ASIA, American Spinal Injury Association; ASA, American Society of Anesthesiologists.

**Table 2:** Summary of perioperative factors and clinical results.

Characteristic		Group A (n=34)	Group B (n=22)	P value
Operative time, mean (SD), minutes		188.1 (74.7)	210.0 (57.4)	0.24

Blood loss, mean (SD), mL		269.8 (421.1)	436.7 (514.4)	0.19

Transfusion, yes, n (%)		6 (17.6)	6 (27.3)	0.29

No. of levels fused, mean (SD)		5.4 (1.9)	5.7 (1.8)	0.53

Posterior decompression, yes, n (%)		12 (35.3)	16 (72.7)	0.006

Perioperative complications, yes, n (%)		4 (11.8)	5 (22.7)	0.23
	Epidural hemorrhage	1	2	
	Massive bleeding (>1500 ml)	0	1	
	peritonitis	0	1	
	Surgical site infection	0	1	
	Instrumentation failure	1	0	
	Deep vein thrombosis	1	0	
	Dural tear	1	0	

Additional adjuvant therapy, yes, n (%)	Chemotherapy	27 (79.4)	10 (45.5)	0.009
	Radiotherapy	19 (55.9)	14 (63.6)	0.56
	Bone modifying agent	26 (76.5)	19 (86.4)	0.36

Postoperative ASIA classification, n (%)	A	1 (2.9)	1 (4.5)	0.15
	B	0	1 (4.5)	
	C	3 (8.8)	4 (18.2)	
	D	8 (23.5)	9 (40.9)	
	E	22 (64.7)	7 (31.8)	

Neurological improvement by at least 1 grade or maintenance of grade E, n (%)		23 (67.6)	9 (40.9)	0.048

Postoperative Barthel index, mean (SD)		87.9 (24.6)	68.8 (38.1)	0.04

Postoperative VAS, mean (SD)		1.6 (1.9)	2.3 (2.0)	0.21

Postoperative course, n (%)	Discharge to home	29 (85.3)	12 (54.5)	0.01
	Transfer to hospice	5 (14.7)	5 (22.7)	0.44
	In-hospital death	0	5 (22.7)	0.004

Revision surgery at local recurrence level, n (%)		1 (2.9)	0	0.60

ASIA, American Spinal Injury Association; VAS, visual analogue scale.

**Table 3:** Neurological recovery in group A based on the ASIA classification.

ASIA classification	Number of patients before surgery	Number of patients after surgery				
		A	B	C	D	E
A	0	0	0	0	0	0

B	1	1	0	0	0	0

C	6	0	0	3	1	2

D	21	0	0	0	7	14

E	6	0	0	0	0	6

Total	34	1	0	3	8	22

ASIA, American Spinal Injury Association.

**Table 4:** Neurological recovery in group B based on the ASIA classification.

ASIA classification	Number of patients before surgery	Number of patients after surgery				
		A	B	C	D	E
A	1	1	0	0	0	0

B	1	0	1	0	0	0

C	6	0	0	3	2	1

D	12	0	0	1	6	5

E	2	0	0	0	1	1

Total	22	1	1	4	9	7

ASIA, American Spinal Injury Association.

## Data Availability

The clinical data used to support the findings of this study are included within the article.
